# Preoperative neutrophil-to-lymphocyte ratio predicts recurrence of patients with single-nodule small hepatocellular carcinoma following curative resection: a retrospective report

**DOI:** 10.1186/s12957-015-0670-y

**Published:** 2015-09-02

**Authors:** Rui Liao, Zhuo-Wei Tang, De-Wei Li, Shi-Qiao Luo, Ping Huang, Cheng-You Du

**Affiliations:** Department of Hepatobiliary Surgery, The First Affiliated Hospital of Chongqing Medical University, Chongqing, 400016 China; Department of General Surgery, Mianyang Central Hospital, Mianyang, 621000 China

**Keywords:** Liver cancer, Inflammation, Neutrophil, Lymphocyte, Prognosis

## Abstract

**Background:**

Preoperative neutrophil-to-lymphocyte ratio (NLR) has been identified as a predictor for the recurrence of hepatocellular carcinoma (HCC), but the cut-off of NLR is inconsistent in various studies. Thus, we detected the prognostic value of preoperative NLR in the single-nodule small HCC (SHCC) patients using X-tile for cutpoint.

**Methods:**

Between January 2007 and December 2010, a total of 222 single-nodule SHCC patients underwent curative resection and were examined for the prognostic roles of preoperative NLR by X-tile.

**Results:**

In this study, all patients were divided into the low-NLR subgroup (NLR ≤ 2.1) and the high-NLR subgroup (NLR > 2.1) by X-tile. Preoperative NLR showed predictive value for time to recurrence (TTR) and overall survival (OS). Moreover, NLR was associated with total bilirubin, white blood cell counts, and HBsAg, respectively (*P* = 0.012, <0.001, and 0.011, respectively). Especially, NLR could discriminate the outcome of patients in the subgroup with alpha-fetoprotein (AFP) levels of ≤400 ng/mL. Importantly, postoperative transcatheter arterial chemoembolization (TACE) had close relationship with OS (*P* = 0.001) and TTR (*P* ≤ 0.001).

**Conclusions:**

Therefore, this study indicates that preoperative NLR, divided by X-tile for the cutpoint, is a simple prognostic marker for the patients with single-nodule SHCC after curative resection.

## Background

Hepatocellular carcinoma (HCC) is a typical inflammation-related malignancy mainly induced by hepatitis B or C viral (HBV or HCV) infection and is the major challenge and cancer burden for China’s health system [1–3]. Surgical resection is still the mainstay of curative treatment. Although surgical techniques and perioperative care are improved recently, the long-term clinical outcomes of HCC remain disappointing owing to a high recurrence rate after surgical resection [4, 5]. To date, many biomarkers have been developed to provide information about the prognosis and treatment of HCC [6–9]. However, serum alpha-fetoprotein (AFP) is still widely used even though normal serum AFP is found in 30 to 40 % of HCC patients [10]. Therefore, it is of great urgent for us to set up reliable and convenient prognostic biomarkers to select optimal candidates to adopt preventive and therapeutic strategies and screen the prognosis of patients at risk.

Recently, there are increasing evidences that metachronous carcinogens are affected by inflammatory activity and the malignant potential of cancer cells are the important causes of HCC recurrence [11, 12]. Notably, accumulating evidences demonstrated that imbalance of the systemic inflammatory response orchestrated a tumor-supporting microenvironment and led to upregulation of the inflammatory process for patients with HCC [13–15], via some molecular pathways related with apoptosis inhibition, DNA damage repair, and angiogenesis promotion [1, 16–19]. Previous studies have revealed inflammatory environment in HCC composed of various inflammatory/immune cells including neutrophil [20], macrophages [21], Treg [22], and Th17 [7] lymphocytes which were closely related with the prognosis of HCC. Consistent with this work, neutrophil-to-lymphocyte ratio (NLR) was also identified as a marker of systemic inflammation response associated with the prognosis of various cancers such as colorectal cancer [23], pancreatic cancer [24], gastric cancer [25], and HCC [26]. Of particular note in HCC, NLR showed good predictive abilities for patients who underwent curative resection [27], liver transplantation [28], transcatheter arterial chemoembolization (TACE) [29], and radiofrequency ablation [30]. To our knowledge, there is no study on the association between the predictive role of NLR and the patients with single-nodule small HCC (SHCC). Additionally, the cut-off value of NLR is not consistent, which is commonly set at five empirically [31], mean value [32], or determined by receiver operating characteristic curve [26] and significant associated hazard ratio [28]. These different selections would hinder the clinical application and comparative study of NLR in different patient populations. Thus, in this study, we used “minimum *P* value” approach [33, 34] by X-tile software, a bio-informatics tool for biomarker assessment, to get an optimal cut-off value of NLR and evaluate the correlation of preoperative NLR with prognosis in single-nodule SHCC patients following curative resection.

## Methods

### Patients and follow-up

A total of 222 patients included in this retrospective cohort study were randomly selected from 256 consecutive patients with pathologically confirmed HCC who were eligible for surgical resection in The First Affiliated Hospital of Chongqing Medical University from 2007 to 2010. Thirty-four patients were excluded according to the following inclusion and exclusion criteria: (1) primary SHCC with a single tumor ≤5 cm, (2) Child-Pugh A liver reserve function, (3) complete laboratory test data, (4) the absence of preoperative extrahepatic metastases, (5) no preoperative anticancer treatments, (6) tumor-free surgical margins, (7) complete patient records and follow-up data, and (8) survival for more than 30 days. The study was approved by the Ethics Committee of The First Affiliated Hospital of Chongqing Medical University, and written informed consents were obtained from all patients.

Sixty-nine patients underwent TACE in 1 month after surgery because they met the eligibility criteria for postoperative TACE in our department as follows: (1) age 18 to 70 years old, (2) Child-Pugh A or B liver function, (3) no severe coagulopathy (platelet count ≥50 × 10^9^/L or prothrombin activity ≥40 %), (4) no obstructive jaundice, and (5) Eastern Cooperative Oncology Group cores ≤2. TACE was carried out by clinicians with more than 10 years of experience. Briefly, a highly selective 5-F catheter was used to assess the arterial blood supply to the liver by visceral angiography. Then, a mixture of three chemotherapeutic agents including 200 mg oxaliplatin, 40 mg epirubicin, and 160 mg irinotecan was used for hepatic artery infusion chemotherapy. And then, 5 mL of Lipiodol was injected. After embolization, the extent of vascular occlusion was determined and blood flow in other arterial vessels was assessed by angiography.

Postoperatively, all patients were followed up every 1–6 months according to the postoperative time with serum alpha-fetoprotein (AFP) and abdominal CT or/and MRI. Tumor recurrences were diagnosed based upon the combined findings of typical CT or MRI appearance and elevated AFP level. Time to recurrence (TTR) [35] was defined as the interval that recurrence was first confirmed after surgery. Overall survival (OS) was the interval from the first operation to death or the latest follow-up visit. The median follow-up time was 42.4 months in these patient populations. Tumor-node-metastasis (TNM) classification system of International Union Against Cancer (edition 7) and tumor characteristics, such as tumor capsule formation and vascular invasion, were assessed as described previously [11].

### Selection of cut-off value for NLR

Here, “minimum *P* value” approach [33, 34] was applied to estimate an optimal cut-off of NLR for the best separation of patients’ TTR by X-tile software [36], version 3.6.1 (Yale University, New Haven, CT). Subsequently, the prognostic significance of the NLR level in single-nodule SHCC was investigated. X-tile software was developed by Robert et al. from Yale University in 2004 [36]. X-tile plots can divide a population into three levels (low, medium, and high level) and separate a single cohort into training and validation subgroups for *P* value estimation. This software shows the users an “on-the-fly” histogram which can provide an associated Kaplan-Meier curve, and the best *P* value is available after rigorous statistical evaluation by X-tile.

### Statistic analysis

Results were presented as the mean ± SD. For the comparison of variables, *t* test, *χ*^2^ test, and Spearman ρ coefficient test were carried out as appropriate, respectively. Medium of age, alanine aminotransferase (ALT), aspartate aminotransferase (AST), total bilirubin (TB), creatinine, platelet counts, neutrophil counts, lymphocyte counts, and white blood cell (WBC) were used as cut-off values. Univariate analysis and multivariate Cox proportional hazards model were used to estimate OS and TTR. All statistical analyses were completed with SPSS 16.0 (SPSS, Inc., Chicago, IL), and a two-tailed *P* value less than 0.05 was considered significant.

## Results

### Baseline characteristics

The baseline characteristics of 222 SHCC patients with single nodule were described in Table 1. Among them, there were 189 men and 33 women with the average age of 53 years. The median follow-up time was 42.4 months. Of the 222 incident of SHCC cases, median tumor size was 3.0 cm. A total of 114 patients had tumor ≤3.0 cm in diameter, and 108 patients had tumor >3.0 cm in diameter. According to the TNM staging system, 44 (19.8 %) patients in our study populations belonged to stage IIIA (44/222). The median values of lymphocyte counts, neutrophil counts, WBC counts, and NLR were 1.5 × 10^9^/L, 2.9 × 10^9^/L, 5.2 × 10^9^/L, and 1.9, respectively. Some laboratory tests were found to be higher than normal values. For example, median values of ALT (normal value is 40 U/L) and AST (normal value is 40 U/L) were 39 and 33 U/L, respectively.

**Table 1:** Characteristics of patients according to NLR

Characteristics	NLR		*P*
	≤2.1 (*n* = 130) >2.1 (*n* = 92)		
Gender (male vs female)	111 vs 19	78 vs 14	NS
Age	52.0 ± 11.3	55.1 ± 10.0	0.049
Albumin (g/L)	41.81 ± 5.13	42.13 ± 4.77	NS
ALT (U/L)	59.88 ± 82.22	64.65 ± 110.44	NS
AST (U/L)	39.25 ± 20.63	54.99 ± 83.46	NS
TB (mg/dL)	0.84 ± 0.34	1.16 ± 1.08	0.012
Cr (mg/dL)	0.87 ± 0.17	0.98 ± 0.78	NS
Lymphocyte counts (10^9^/L)	1.80 ± 0.66	1.20 ± 0.50	<0.001
Neutrophil counts (10^9^/L)	2.59 ± 0.83	4.70 ± 2.61	<0.001
WBC counts (10^9^/L)	4.90 ± 1.45	6.49 ± 2.79	<0.001
HBsAg (positive vs negative)	118 vs 12	72 vs 20	0.011
Anti-HCV (positive vs negative)	1 vs 129	0 vs 92	NS
AFP (ng/mL) (≤20 vs >20)	51 vs 79	40 vs 52	NS
INR	1.02 ± 0.077	1.03 ± 0.09	NS
Platelet counts (10^9^/L)	137.36 ± 57.08	134.16 ± 62.42	NS
Vascular invasion (yes vs no)	25 vs 105	24 vs 68	NS
Tumor differentiation (I–II vs III–IV)	94 vs 36	73 vs 19	NS
Tumor encapsulation (yes vs no)	50 vs 80	45 vs 47	NS
Tumor size (≤3.0 vs >3.0)	74 vs 56	40 vs 52	NS
TNM stage (I–II vs IIIA)	108 vs 22	70 vs 22	NS
Postoperative TACE (yes vs no)	37 vs 93	32 vs 60	NS
Re-operation (yes vs no)	10 vs 120	6 vs 86	NS

*NLR* neutrophil-to-lymphocyte ratio, *ALT* alanine aminotransferase, *AST* aspartate aminotransferase, *TB* total bilirubin, *Cr* creatinine, *WBC* white blood cell, *HBsAg* hepatitis B surface antigen, *AFP* alpha-fetoprotein, *INR* international normal ratio, *TNM* tumor-node-metastasis, *TACE* transarterial chemoembolization

### A best cut-off value for NLR

**Fig. 1:**
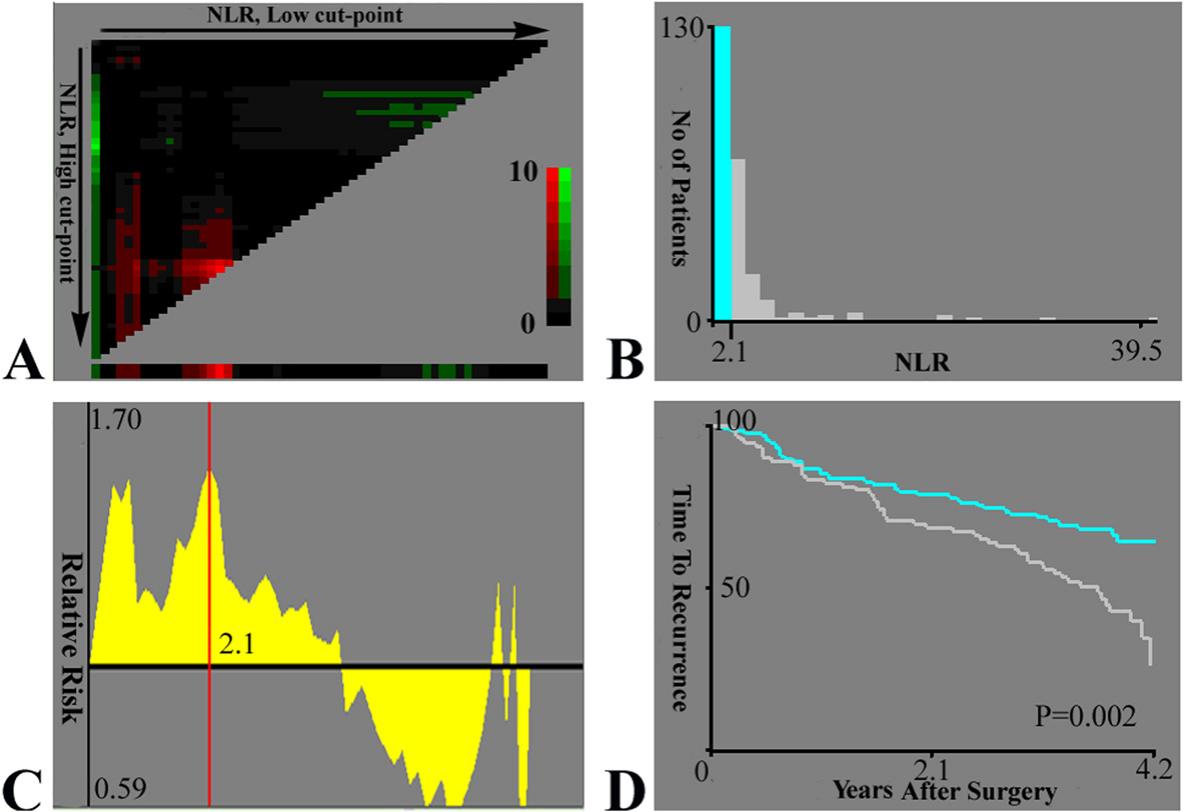
X-tile plots of NLR on single-nodule small hepatocellular carcinoma following curative resection. The X-tile plots show the *χ* ^2^ log-rank values with cutpoint, producing low and high subgroups. **a** The *X*-axis represents all potential cut-off values from low to high (*left to right*) that define a low subset, whereas the *Y*-axis represents cut-off values from high to low (*top to bottom*) that define a high subset. *Red coloration* of cut-off values indicates an inverse correlation with time to recurrence, and *green coloration* represents direct associations. The optimal cut-off value occurs at the brightest pixel (*green* or *red*). **b** A histogram of the entire cohort divided into low and high subgroups according to the optimal cut-off value of NLR (2.1). **c** The statistical significance of relative risk (*RR*) generally assessed by Cox proportional hazards model. The *X*-axis represents all potential cut-off values from low to high (*left to right*), and the *Y*-axis of the graph is log of the relative risks. The *red line* is the optimal cutpoint of NLR (2.1). **d** A Kaplan-Meier plot of NLR for time to recurrence produced by the optimal cut-off value of NLR. *Blue* represents the low subgroup, and *gray* represents the high subgroup

According to the “minimum *P* value” approach, X-tile software was applied to estimate the optimal cut-off of NLR for the best separation of patients’ TTR (Fig. 1). Here, *P* = 0.002 was minimum *P* value when NLR arrived at 2.1, which subsequently was identified as the best cut-off point of NLR for operative recurrence. Accordingly, all the patients were divided into two groups: a low-NLR group (≤2.1, *n* = 130) and a high-NLR group (>2.1, *n* = 92, Table 1).

### Correlation of NLR with clinicopathologic features

As shown in Table 1, preoperative NLR was associated with some clinical pathologic characteristics. Old SHCC patients had higher incidence to have elevated NLR (*P* = 0.049). Moreover, NLR (low vs high) was related with several laboratory tests rather than tumor factor, such as TB, WBC, and HBsAg (*P* = 0.012, <0.001 and 0.011, respectively). No obvious correlation with gender, ALT, AST, TB, creatinine, and platelet counts was observed (all *P* > 0.05).

### NLR was an independent prognostic factor

**Fig. 2:**
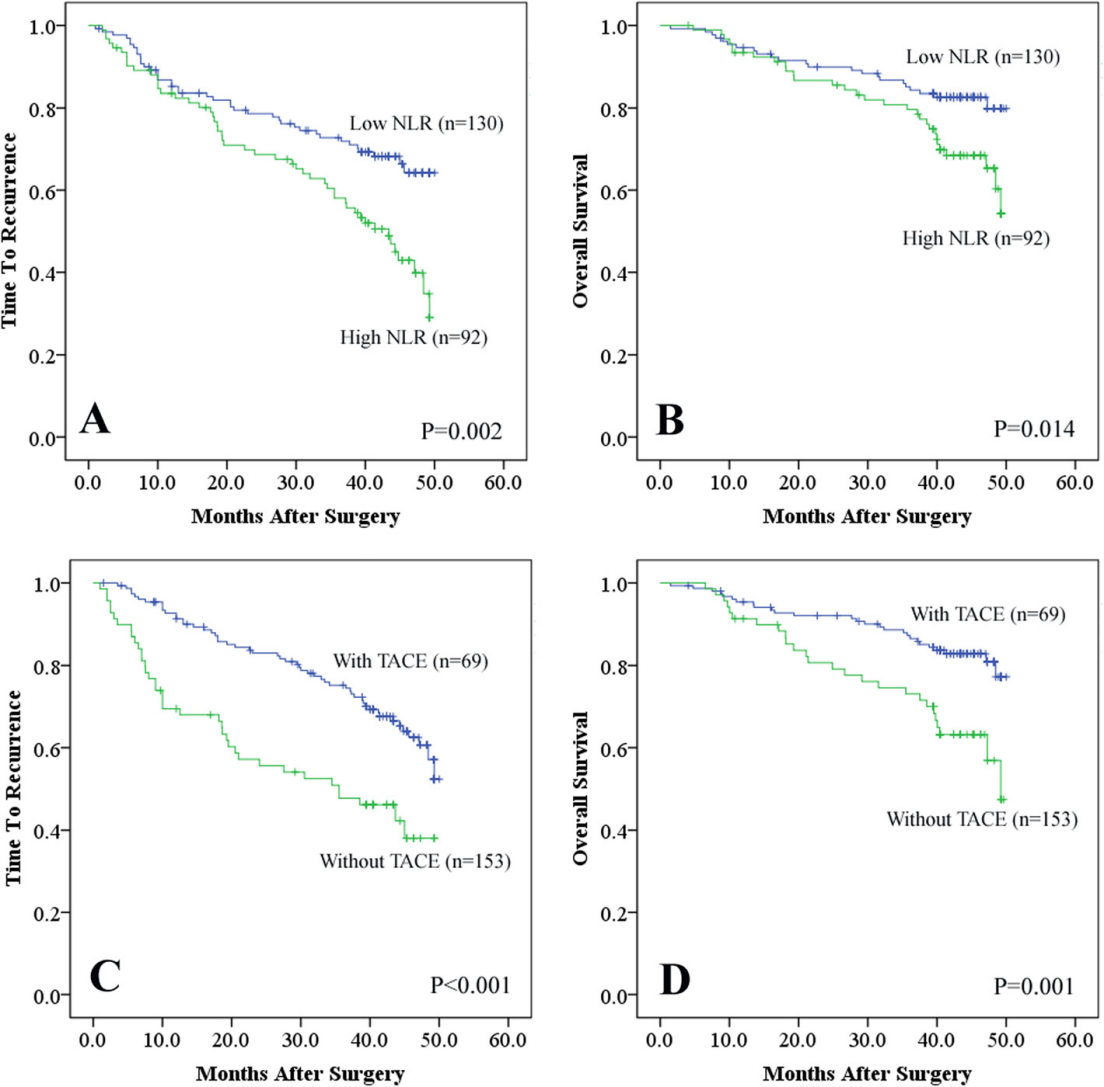
Prognostic values of preoperative neutrophil-to-lymphocyte ratio (*NLR*) and postoperative transcatheter arterial chemoembolization (*TACE*). **a**, **b** Kaplan-Meier estimates of time to recurrence (*TTR*) and overall survival (*OS*) of NLR which were divided into low (NLR ≤ 2.1) and high (NLR > 2.1) subgroups, respectively. **c**, **d** TTR (**c**) and OS (**d**) of patients with or without postoperative TACE by Kaplan-Meier analyses, respectively

The 1-, 3-, and 5-year OS of these single-nodule SHCC patients were 94.1, 82.9, and 65.4 %, respectively, and the 1-, 3-, and 5-year TTR rates were 85.4, 66.7, and 45.9 %, respectively. On univariate analyses of our data, international normal ratio (INR), platelet counts, and neutrophil counts were demonstrated to be related with OS (*P* = 0.010, 0.046, and 0.040, respectively). Old age, AFP, vascular invasion, and re-operation showed association with TTR (*P* = 0.004, 0.043, 0.001, and <0.001, respectively). Moreover, tumor size, postoperative TACE, and preoperative NLR had prognostic significance for both OS (*P* = 0.040, 0.001, and 0.014, respectively) and TTR (*P* < 0.001, <0.001, and 0.002, respectively, Table 2 and Fig. 2).

**Table 2:** Prediction of survival and recurrence of patients with a single nodule of SHCC following curative resection

Factors	OS			TTR		
	Univariate multivariate			Univariate multivariate		
	*P*	HR (95 % CI)	*P*	*P*	HR (95 % CI)	*P*
Age (≤53 vs >53)	NS		NA	0.004		NS
AFP(≤20 vs >20)	NS		NA	0.043		NS
INR (≤1.0 vs >1.0)	0.010		NS	NS		NA
PLT counts (≤127 vs >127)	0.046		NS	NS		NA
Neutrophil counts	0.040	0.329 (0.174–0.623)	0.001	NS		NA
Vascular invasion (yes vs no)	NS		NA	0.001	1.757 (1.110–2.781)	0.016
Tumor size (≤3.0 vs >3.0)	0.040		NS	<0.001		NS
Postoperative TACE (yes vs no)	0.001	2.566 (1.488–4.424)	0.001	<0.001	2.175 (1.408–3.359)	<0.001
Re-operation (yes vs no)	NS		NA	<0.001	9.037 (4.970–16.432)	<0.001
NLR (>2.1 vs ≤2.1)	0.014	3.013 (1.633–5.561)	<0.001	0.002	1.619 (1.057–2.478)	0.027

*Univariate analysis* Kaplan-Meier method, *Multivariate analysis* Cox proportional hazards regression model, *SHCC* small hepatocellular carcinoma, *AFP* alpha-fetoprotein, *INR* international normal ratio, *PLT* platelet, *TACE* transarterial chemoembolization, *NLR* neutrophil-to-lymphocyte ratio, *OS* overall survival, *TTR* time to recurrence, *HR* hazard ratio, *NS* not significant, *NA* not adopted

**Fig. 3:**
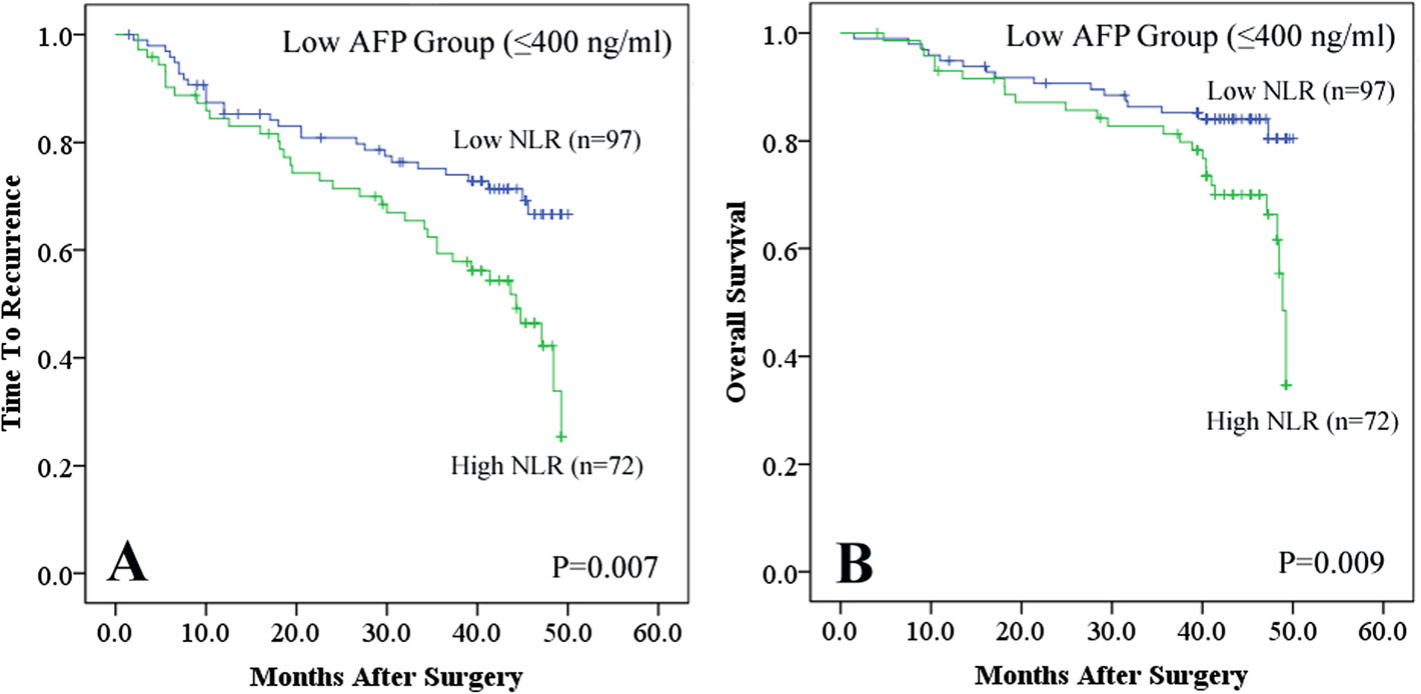
Kaplan-Meier analyses of neutrophil-to-lymphocyte ratio (*NLR*) in the low AFP subgroup (≤400 ng/mL). NLR was divided into low (NLR ≤ 2.1) and high (NLR > 2.1) subgroups by X-tile. In the low AFP subgroup (≤400 ng/mL), NLR could predict time to recurrence (**a**) and overall survival (**b**), respectively

Then, multivariate analyses were used to examine the association between significant clinical factors and NLR. Neutrophil counts showed higher predictive value on OS (*P* = 0.001). Vascular invasion and re-operation were all independent predictors for TTR (*P* = 0.016 and <0.001, respectively). Importantly, both postoperative TACE and preoperative NLR had close relationship with OS (*P* = 0.001 and <0.001, respectively) and TTR (*P* = <0.001 and 0.027, respectively). In addition, the prognostic significance of preoperative NLR also applied to SHCC patients with low AFP levels of ≤400 ng/mL by stratified analyses (*P* = 0.007 for TTR and 0.009 for OS, respectively, Fig. 3).

## Discussion

Cancer is characterized by complex tissues closely related with chronic inflammatory contributing to progressive development and high postoperative recurrence risk [1]. There are increasing evidences that systemic inflammation is responsible for the tumor-promoting activities and associated with the recurrence of certain tumors [16, 17]. Recently, as a typical systematic inflammatory biomarker, NLR has also been identified as a predictor for the outcomes of various tumors [23–26]. However, the reported cut-off values of NLR were different in various studies, which could not provide a consistent standard for comparison among different patient populations. Therefore, for the first time, we used X-tile software, a bio-informatics tool especially for biomarker assessment, to determine the cut-off value of NLR in single-nodule SHCC patients. In this study, we clearly observed that using X-tile, high preoperative NLR could predict the poor survival of SHCC patients with single nodule after hepatectomy. This result revealed that even in small and single tumor, systematic inflammatory responses play critical roles and exist along the path of HCC progression. Moreover, we also found that postoperative TACE could improve the outcomes of patients with single-nodule SHCC. However, the choice of postoperative TACE could bias the survival outcomes because it is not a randomized procedure, and the further randomized controlled trial with larger sample size from multicenter is necessary.

X-tile software was developed by Robert et al. [36], aiming to assess the biological relationships between biomarkers and outcomes of certain diseases. This tool could produce corrected *P* values to evaluate statistical significance of data assessed by multiple cutpoints. Consistent with previous applications [7, 9], X-tile produced an optimal cut-off of NLR which presents an accurate prognostic factor in cases of SHCC with single nodule. We believe that there is no unique preoperative NLR that satisfies all patient populations of the world. X-tile could provide objective and accurate cut-off of NLR to investigate its association with the recurrence of single-nodule SHCC.

In the present study, we found that NLR was associated to some laboratory parameters, such as TB (*P* = 0.012), HBsAg (*P* = 0.011), and WBC (*P* < 0.001). On one hand, the close relationship between NLR and WBC demonstrated that NLR was an inflammation-related biomarker and could accurately reflect an inflammatory status. On the other hand, as well known, one of the typical features of HCC is a high frequency of hepatitis B or C viral (HBV or HCV) infection and subsequently fibrosis or cirrhosis. As mentioned above, high NLR reflects aggravated inflammatory status of SHCC patients, majority of who have poor liver function and high load of hepatitis viral infection. Therefore, to some extent, TB and HBsAg could respond to this disease condition. Although there was no association between NLR and tumor factors, we thought that it was the reason of the selection of patients who all were single-nodule SHCC and had better tumor differentiation (I–II vs III–IV, 167 vs 55) and earlier tumor stage (I–II vs IIIA, 178 vs 44). After all, among these patients, tumor-promoting inflammation would overwhelm antitumor immunity with the development of tumor.

Although the precise tumor-promoting inflammatory mechanisms related with NLR remain to be clear, our previous studies and other investigations could provide several possible explanations for the predictive value of NLR. First, previous study found that intratumoral neutrophils could serve as a predictor for HCC recurrence [20]. This result was consistent with other reports that neutrophils promoted tumor invasion and formation of hepatic metastasis via mutual interactions with HCC cells [37] and circulating tumor cells [38]. We therefore assume that neutrophil-mediated mechanisms are involved in extracellular matrix remodeling [20], growth factors [20], and initial angiogenic switch [39] contributing to reroute the inflammatory response into a tumor-promoting direction. Second, elevated NLR usually represents relative lymphocytopenia. As we know, the host’s anticancer immune response greatly depends on lymphocytes. Various types of lymphocytes such as intratumoral regulatory T cells [22], Th17 cells [7], and gammadelta T cells [40] constituted the immunosuppressive network within the tumor milieu and may promote proliferation and metastatic activity of tumor cells by the roles in cytotoxic cell death and cytokine production [41]. Third, increased NLR may stand for the imbalance between tumor-promoting inflammation (neutrophils) and antitumor immunity (lymphocytes), so that systemic inflammatory response has an overrepresentation, thereby lead to a much worse prognosis for patients with SHCC. This present study also revealed that many patients with elevated NLR have high level of WBC, also showing the critical role of inflammatory response in tumor development. Interestingly, a study built on a mouse model demonstrated that naturally activated neutrophils (polarized N2 phenotype) promoted tumor progression partly related with suppression of CD8^+^ T cells [42]. However, studies in depth are needed to reveal the molecular mechanisms of NLR in SHCC development.

Here, we did not find the relationship between preoperative NLR and early (≤24 months) or later recurrence (>24 months), probably because the follow-up period was relatively short and sample size was small. Another explanation may be the effect of inflammatory response in single-nodule SHCC was obstructed in time by surgery and various treatments before it spread out completely. Moreover, many clinical studies demonstrated that low AFP levels of ≤400 ng/mL were a significant favorable prognostic factor for HCC. Nevertheless, 30–40 % of HCC patients with low serum AFP concentration had recurrence and were difficult to monitor [43–45]. We found that preoperative NLR had the ability to discriminate patients with worse survival and higher recurrence rates even in the subgroup with lower AFP levels of ≤400 ng/mL [44, 46]. Therefore, single-nodule SHCC patients with higher preoperative NLR and lower AFP (≤400 ng/mL) still require closer follow-up since they have more possibilities to suffer from tumor recurrence. But, AFP >400 ng/mL was not shown any association with the poor outcomes of patients with single-nodule SHCC undergoing curative resection. Considering the relative small sample size (*n* = 53 for the patients with AFP >400 ng/mL), a conclusion about the proangiogenic role of low AFP levels of >400 ng/mL in single-nodule SHCC should be cautiously interpreted.

Thus, we believe that in the future, larger patient population or a validation cohort with longer follow-up period could prove it as a clinically useful or applicable finding for patients with single-nodule SHCC treated with curative resection. Additionally, some biomarkers were identified as good candidates of inflammatory markers such as C-reactive protein, Treg, and Th17 lymphocytes. Therefore, further comparative study in HCC would make us better understand the predictive value of NLR as an inflammatory index if complete data could be obtained.

## Conclusions

In conclusion, we have shown that preoperative NLR, divided by X-tile for the cutpoint, is a simple biomarker of systemic inflammatory response and unfavorable prognosis of single-nodule SHCC after curative resection. Although we also found that postoperative TACE could improve the outcome of patients with single-nodule SHCC, the further randomized controlled trial with larger sample size from multicenter is necessary.
